# Selection and Spread of Artemisinin-Resistant Alleles in Thailand Prior to the Global Artemisinin Resistance Containment Campaign

**DOI:** 10.1371/journal.ppat.1004789

**Published:** 2015-04-02

**Authors:** Eldin Talundzic, Sheila Akinyi Okoth, Kanungnit Congpuong, Mateusz M. Plucinski, Lindsay Morton, Ira F. Goldman, Patrick S. Kachur, Chansuda Wongsrichanalai, Wichai Satimai, John W. Barnwell, Venkatachalam Udhayakumar

**Affiliations:** 1 Malaria Branch, Division of Parasitic Diseases and Malaria, Center for Global Health, Centers for Disease Control and Prevention, Atlanta, Georgia, United States of America; 2 Atlanta Research and Education Foundation, Atlanta VA Medical Center, Atlanta, Georgia, United States of America; 3 Bureau of Vector Borne Diseases, Ministry of Public Health, Nonthaburi, Thailand; 4 Bansomdej-chaopraya Rajabhat University, Bangkok, Thailand; 5 Independent Scholar, Bangkok, Thailand; National Institute of Allergy and Infectious Diseases, UNITED STATES

## Abstract

The recent emergence of artemisinin resistance in the Greater Mekong Subregion poses a major threat to the global effort to control malaria. Tracking the spread and evolution of artemisinin-resistant parasites is critical in aiding efforts to contain the spread of resistance. A total of 417 patient samples from the year 2007, collected during malaria surveillance studies across ten provinces in Thailand, were genotyped for the candidate *Plasmodium falciparum* molecular marker of artemisinin resistance K13. Parasite genotypes were examined for K13 propeller mutations associated with artemisinin resistance, signatures of positive selection, and for evidence of whether artemisinin-resistant alleles arose independently across Thailand. A total of seven K13 mutant alleles were found (N458Y, R539T, E556D, P574L, R575K, C580Y, S621F). Notably, the R575K and S621F mutations have previously not been reported in Thailand. The most prevalent artemisinin resistance-associated K13 mutation, C580Y, carried two distinct haplotype profiles that were separated based on geography, along the Thai-Cambodia and Thai-Myanmar borders. It appears these two haplotypes may have independent evolutionary origins. In summary, parasites with K13 propeller mutations associated with artemisinin resistance were widely present along the Thai-Cambodia and Thai-Myanmar borders prior to the implementation of the artemisinin resistance containment project in the region.

## Author Summary

The *Plasmodium falciparum* parasites that cause malaria are evolving resistance to our most effective and potent anti-malarial drugs available, called artemisinins. Currently, artemisinin resistance is emerging in a number of countries in the Greater Mekong Subregion, including Cambodia, Thailand, Myanmar, and Vietnam. Historically, the Thai-Cambodia border region has been an epicenter of resistance to several anti-malarial drugs. To prevent the spread of artemisinin resistant parasites from the Greater Mekong Subregion, a global artemisinin resistance project was initiated in 2009. Here, we show that artemisinin resistance associated mutation in the K13 gene were widely present throughout Thailand, as early as 2007, primarily along the Thai-Cambodia and Thai-Myanmar border regions. Additional data based on microsatellite markers suggests that the most commonly found K13 C580Y allele may have two recent independent origins in Thailand, on the borders of Cambodia and Myanmar.

## Introduction

Artemisinin combination therapy (ACT) has been adopted globally as the first-line treatment for uncomplicated *Plasmodium falciparum* malaria and has contributed to the reduction in malaria related mortality and morbidity. However, resistance to artemisinin poses a threat to the global effort to control malaria. In 2008–2009 the first verified cases of artemisinin resistance, characterized by delayed parasite clearance, were observed in western Cambodia [1]. Prior to those cases, instances of reduced parasite susceptibility to artemisinin were reported in parts of Thailand bordering Cambodia (Chantaburi, Trat, Sakaew, Sisaket, Burirum, and Surin provinces) and Myanmar (Tak province) as early as 2003 [2]. The Thai-Cambodian border region has historically been the epicenter of multi-drug resistant (MDR) malaria [3]. As resistance to earlier anti-malarial drugs spread from this region to Africa and other parts of Asia through parasite migration, there is a serious concern that a similar scenario may occur with artemisinin resistance [4]. The ACT artesunate-mefloquine (ASMQ) has been used as first-line therapy in Thailand since 1995, beginning in areas where multi-drug resistance had evolved. Use of ASMQ was extended to the rest of the country after the World Health Organization (WHO) recommended ACT for global use in the early 2000s [5]. In Thailand, ASMQ was initially introduced as a two-day regimen and in 2008 was extended to three days (three days of artesunate and two days of mefloquine, but with the same total dose as the two-day regimen) [6].

The ASMQ regimen has remained generally effective in Thailand despite high levels of mefloquine resistance with a cure rate of greater than 90% [5]. However, in several locations in Cambodia and Thailand, treatment failure rates over 10% have also been observed [7]. To date, the strongest evidence of artemisinin resistance was initially reported in western Cambodia, and subsequently other parts of Southeast Asia [1, 7–13].

To prevent the spread of artemisinin-resistant *P*. *falciparum*, the WHO and other partners initiated an artemisinin resistance containment project for the Greater Mekong Subregion in 2009 [14]. The goal was to identify and prevent artemisinin-resistant parasites from spreading outside of documented hotspot regions along the Thai-Cambodian border by ensuring proper diagnosis and full treatment of reported malaria cases [15]. Subsequently, the WHO, along with other partners, initiated the Global Plan for Artemisinin Resistance Containment and Emergency Response to artemisinin resistance in the Greater Mekong Subregion [5].

Currently, therapeutic efficacy studies (TES) are considered the gold standard for determining antimalarial drug efficacy [16]. However, the WHO recommends that TES results be complimented using molecular marker studies [17]. Therefore, it was desirable to identify a molecular marker for artemisinin resistance. Initial studies using a genome-wide association approach found two loci on *P*. *falciparum* chromosomes 10 and 13 to be associated with artemisinin resistance [12, 18]. After a long search to pinpoint a specific gene associated with artemisinin resistance, the K13 gene (*PF3D7_1343700*) on chromosome 13 was identified as a potential molecular marker [19]. The study identified mutations in the propeller domain of the K13 gene that were associated with artemisinin resistance as measured by *ex vivo* ring stage survival assays and delayed parasite clearance times [19]. Specifically, the study identified 18 non-synonymous single nucleotide polymorphisms (SNPs) in the K13 propeller domain, of which three mutations (C580Y, R539T and Y493H) were strongly associated with increased ring stage survival and delayed parasite clearance rates. The C580Y allele accounted for about 85% of all mutant K13 alleles observed in 2011–2012 in western Cambodia [19]. Most recently, a two year multi-site project by Ashley *et al*. further confirmed that multiple SNPs in the propeller domain of K13 were predictive of slow parasite clearance and that these mutations were found in multiple countries in the Greater Mekong Subregion [20]. That study, along with two other recent studies by Takala-Harrison *et al*. and Miotto *et al*. [13, 21], showed that the C580Y allele was the predominant allele in Cambodia, Myanmar, and Vietnam. The authors further demonstrated that the C580Y allele may have emerged independently in Cambodia and Myanmar.

Although Thailand has historically been an epicenter of resistance to several antimalarial drugs, currently there are limited data on the artemisinin resistance-associated K13 propeller mutations in this region. Here, using historical samples collected during 2007 from ten different sites in Thailand, we set forth to answer the following questions: (1) Were artemisinin resistance-associated K13 mutant alleles present in Thailand prior to the implementation of the artemisinin resistance containment projects? (2) If so, what were the prevalence and distribution of the K13 propeller mutations in Thailand? (3) What are the evolutionary histories of the different K13 mutant and wild type alleles? (4) Are the K13 mutant alleles evolving locally or are particular mutants spreading across Thailand? and (5) Is there evidence for selection of resistant K13 alleles?

## Results

### Distribution and frequency of K13 propeller mutations in Thailand during 2007

**Fig 1:**
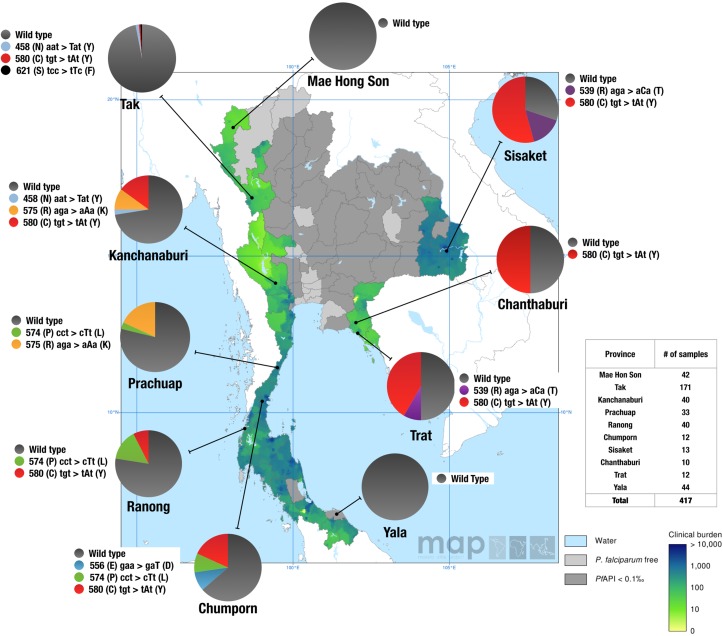
Geographic distribution of the K13 propeller alleles in Thailand in 2007. Pie charts show K13 propeller allele frequencies among 417 parasite isolates in 10 Thailand provinces. The different alleles are color coded. The results are shown on top of the clinical burden map of *P*. *falciparum* in Thailand in 2007 (Malaria Atlas Project) [34]. Light grey areas are *P*. *falciparum* malaria free and dark grey areas have an unstable risk of malaria transmission (i.e. annual case incidence, or API, is reported at less than 1 per 10,000). Map shows mean estimate for the clinical burden in the range from 0 (light green) to 10,000 (dark green/blue) clinical cases per year. The clinical burden predictions are based on a Bayesian geostatistical model.

All 417 patient samples were either wild type or had a single mutation in the K13 propeller domain. Twelve percent (50/417) carried one of seven mutant alleles (N458Y, R539T, E556D, P574L, R575K, C580Y, S621F) in the K13 propeller domain, including two mutations (R575K and S621F) that have not been reported previously in Thailand. The C580Y mutant allele, which is a predominant allele in Cambodia, accounted for 52% (26/50) of all mutant alleles identified in our study population. The C580Y allele frequencies were higher along the Thai-Cambodian border, in Chanthaburi (N = 5/10, 50% C580Y), Trat (N = 5/12, 42% C580Y), and Sisaket (N = 8/13, 62% C580Y) provinces compared to the provinces along the Thai-Myanmar border, Chumporn (N = 2/12, 17% C580Y), Ranong (N = 3/40, 8% C580Y), Kanchanaburi (N = 6/40, 15% C580Y), and Tak (N = 1/171, 1% C580Y). Interestingly, the R539T alleles were only found in eastern Thailand near the Cambodian border in Trat (N = 1/12, 8% R539T) and Sisaket (N = 2/13, 16% R539T) provinces. Besides the C580Y mutation, four previously identified mutations (R575K, P574L, E556D, and N458Y) as well as one novel K13 propeller allele not reported yet (S621F), were found in western parts of Thailand. The R575K and S621F alleles were only present along the Thai-Myanmar border in Prachuap (N = 6/33, 18% R575K), Kanchanaburi (N = 4/40, 10% R575K), and Tak (N = 1/132, 1% S621F), provinces. The P574L allele was present in Ranong (N = 4/40, 10%), followed by 8% (N = 1/12) prevalence in Chumporn and 3% (N = 1/33) in Prachuap. All parasite isolates from the northwestern province of Mae Hong Son (N = 42/42) and southeastern province of Yala (N = 40/40) carried the wild type K13 propeller allele. Overall, these results show the presence of parasites harboring single non-synonymous mutations in the K13 propeller domain as early as 2007 in eight Thai provinces (Fig 1).

### Emergence and spread of the C580Y allele in Thailand

**Fig 2:**
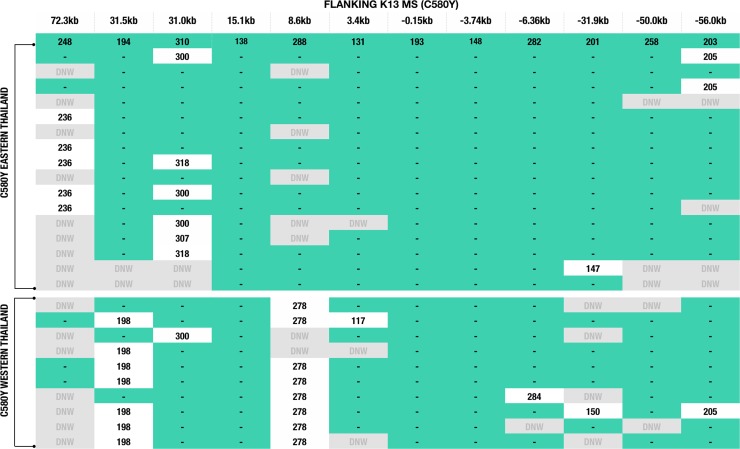
K13 flanking microsatellites of parasites from Thailand. The figure comprises data from 16 western and 10 eastern Thai parasites with the C580Y mutation. Allele lengths are shown for 12 microsatellites positioned at: 72.3kb, 31.5kb, 31.0kb, 15.1kb, 8.6kb, 3.4kb, -0.15kb, -3.74kb, -6.36kb, -31.9kb, -50.0kb, and -56.0kb. Teal green shading and lines indicate identical allele sizes. DNW (in grey) = indicates no successful amplification after a third attempt or not enough DNA was available to repeat the analysis.

Microsatellites flanking K13 were used to infer the evolutionary histories of the C580Y alleles. Parasites with the C580Y alleles from eastern and western Thailand shared a similar genetic profile in most loci, with the exception of the 8.6kb locus downstream of the gene (Fig 2). This microsatellite locus clearly separates the C580Y alleles based on geography (east versus west). Moreover, the C580Y alleles in the eastern region had a 194 bp allele size at locus 31.5kb, whereas in the western Thailand most of the C580Y alleles had a 198 bp allele size with the exception of three isolates (Fig 2). In addition, there is a high degree of microsatellite identity among infections with the C580Y allele as compared to the wild type K13 haplotypes both upstream and downstream from K13 (S1 Dataset).

### Reduced genetic heterozygosity of the C580Y K13 propeller allele

**Fig 3:**
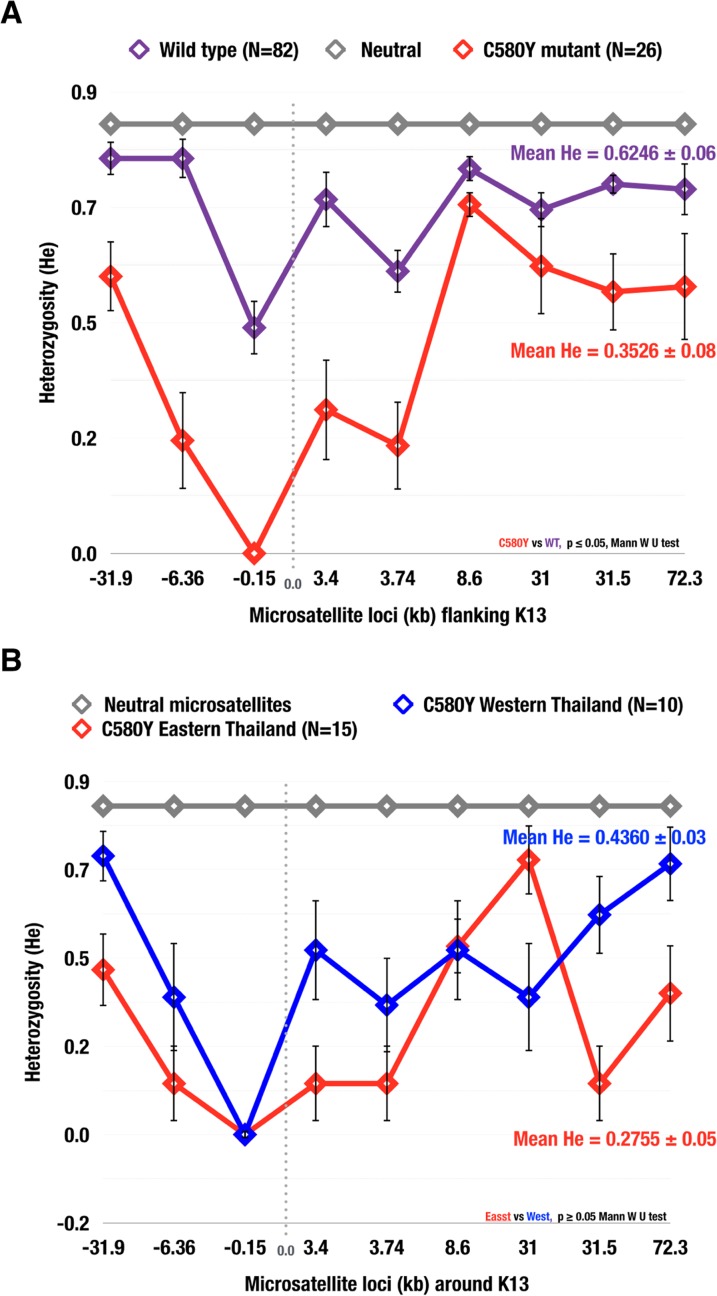
Heterozygosity valley around K13 propeller alleles in Thailand. The expected heterozygosity of parasite isolates with: (A) the C580Y mutation (N = 26) and wild type alleles (N = 82); (B) the C580Y from the east (N = 10) and west (N = 16). Diversity was reduced at all 9 K13 propeller microsatellite loci for C580Y compared to wild type alleles. The mean H*e* in (A) for C580Y (0.3526 ± 0.08), wild type (0.6246 ± 0.06), neutral (0.7650 ± 0.05); (B) C580Y east (0.2755 ± 0.05) and C580Y west (0.4360 ± 0.03 ± 0.03). The error bars indicate ± standard deviation (SD).

Using the nine microsatellite loci flanking the K13 propeller gene, expected heterozygosity (H*e*) was calculated for the C580Y and wild type alleles (Fig 3). The N458Y, R539T, P574L, R575K and S621F alleles were excluded as there were limited samples to carry out the analysis. The C580Y allele (N = 26, mean H*e* = 0.3526 ± 0.08) showed a 56% reduction (p = 0.0046) in heterozygosity as compared to wild type alleles (N = 22, mean H*e* = 0.6246 ± 0.06) (Fig 3A). No significant difference (p = 0.2240) in heterozygosity was found when comparing western C580Y alleles (N = 10, mean H*e* = 0.4360 ± 0.03) to eastern C580Y alleles (N = 15, mean H*e* = 0.2755 ± 0.05) (Fig 3B). Mean H*e* between the wild type and different mutant alleles were compared using the Mann-Whitney U test.

### Principal component analysis and regional separation of C580Y alleleic haplotypes

**Fig 4:**
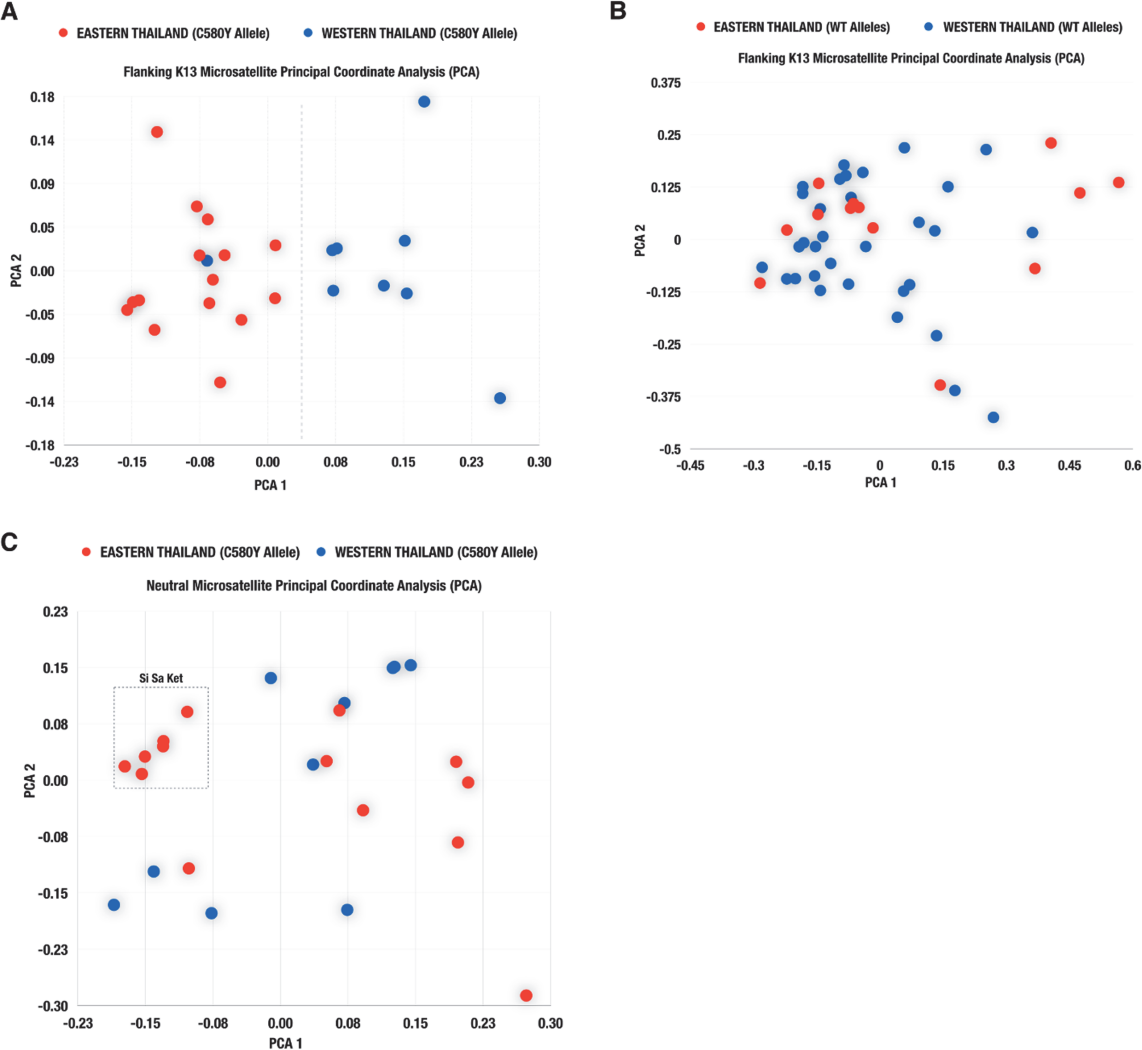
Population structure by geography. Scatter plots from principal component analysis (PCA) based on flanking K13 microsatellite analysis of parasite isolates with the C580Y allele (A) and wild type allele (B). Results of PCA based on neutral microsatellites for parasite isolates carrying the C580Y allele, are shown in (C).

The results of the principal component analysis of the neutral and flanking microsatellite data for the C580Y mutants and wild type isolates are plotted in Fig 4. When considering the flanking microsatellite data for all C580Y mutants, the first principal component separated the isolates into those from the western (Kanchanaburi, Ranong, and Chumporn) and eastern (Chanthanburi, Trat, and Sisaket) provinces (Fig 4A), with the exception of a single isolate. No clear geographical separation is observed when looking at the flanking K13 microsatellites in the wild type population (Fig 4B). Interestingly, some of the parasites from Sisaket Province clustered together suggesting a recent clonal expansion of these parasites (Fig 4C). Similar results were obtained by a neighbor joining tree analysis (S1 Fig).

### Association of genetic dissimilarity with genetic distance

**Fig 5:**
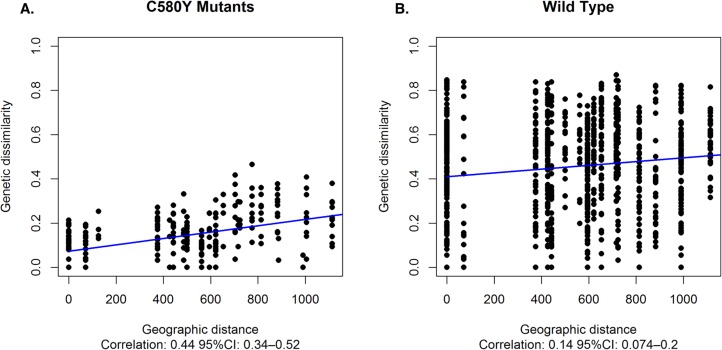
Association between genetic dissimilarity and geographic distance. (A) Genetic dissimilarity and geographic distance between pairs of C580Y mutants (A) and wild type parasites (B). Genetic dissimilarity of flanking microsatellites for C580Y is highly associated with geographic distance (Pearson's correlation coefficient: 0.44, 95% CI:0.34–0.52). Average genetic dissimilarity is lower between C580Y and wild type parasites (t-test p-value < 0.001).

The genetic dissimilarity between the K13 flanking microsatellites of C580Y mutants was highly associated (Pearson's correlation coefficient: 0.44, 95% CI: 0.34–0.52) with the geographic distance between the sites where the isolates were collected. Average genetic dissimilarity was lower between pairs of C580Y mutants than between pairs of wild type parasites (t-test p-value < 0.001), and the association between genetic dissimilarity and geographic distance was stronger for C580Y mutants than for wild type parasites (Fig 5).

## Discussion

To our knowledge, this study is one of the first reports to systematically analyze the artemisinin resistance K13 propeller mutations and flanking microsatellite loci in parasites collected in numerous sites in Thailand shortly before the implementation of the artemisinin resistance containment project in 2009. The samples were collected from across ten provinces, including the containment zones and areas at highest risk for malaria. This study provides evidence that (1) artemisinin resistance alleles were present in 8 out of 10 Thai provinces sampled, including two mutant alleles (R575K and S621F) not previously reported in Thailand, (2) artemisinin resistance-associated K13 alleles had evolved along the Thai-Cambodia and Thai-Myanmar border regions at least two years prior to the implementation of the artemisinin resistance containment project, (3) the artemisinin resistance-associated C580Y mutant alleles were the most common and widespread in Thailand, (4) there are clear differences in microsatellites that differentiate the C580Y mutant alleles from eastern and western parts of Thailand, and (5) the C580Y alleles appear to have had two recent, independent origins.

Our study provides insight into the prevalence and distribution of K13 mutations in Thailand as early as 2007. Interestingly, the prevalence of mutant K13 alleles reported in Pailin, Cambodia during 2007 for the C580Y (45%) and R539T (5%) alleles [19] is consistent with our findings. Across the border from Pailin, in the provinces of Chanthaburi, Sisaket and Trat, we observed that more than 42% of the parasites screened carried the C580Y mutant allele (Fig 1). The C580Y allele has since been associated with delayed parasite clearance in this region [19, 20]. The highest prevalence of the C580Y (62%) and R539T (16%) mutations is seen in the province of Sisaket, just north of Cambodia. These results are in agreement with the recent study by Ashley *et al* [20], which provided compelling evidence that the C580Y and R539T mutations are associated with delayed parasite clearance in both Sisaket and Ranong. Moreover, 78% (7/9) of the parasite isolates from Sisaket showed a similar clonal genetic profile, suggesting that this may have been a recent clonal expansion event (Figs 3C and S1). Similar findings were reported by Miotto *et al* [22], who demonstrated that three subpopulations associated with clinical resistance to artemisinin may have recently expanded in Cambodia and elsewhere in the region.

In eastern Thailand, C580Y and R539T were the only mutations observed; however, this may be due to the limited number of samples analyzed. In contrast, the provinces bordering Myanmar had the following mutations: S621F, C580Y, R575K, P574L, E556D, and N458Y. C580Y, P574L and R575K were the most commonly found alleles along the Thai-Myanmar border region (i.e. from Kanchanaburi to Chumporn). Interestingly, the C580Y and R575K mutations have recently been reported near the Thai-Myanmar border as well [23, 24]. Other mutant alleles (N458Y, S621F, and E556D) were rare and restricted to one or two sites (Fig 1), suggesting that these K13 mutations may have arisen independently. Although previous studies have confirmed a strong association between select K13 propeller domain mutations and delayed parasite clearance [19, 20, 25], it remains to be determined whether the remaining mutant alleles will have a similar association. Furthermore, it remains to be seen whether the C580Y mutation will trend towards fixation in Thailand, as was seen between 2001 and 2012 in Pailin, Cambodia [19]. Our data show that artemisinin-resistant K13 alleles did not spread to or evolve in the southernmost Yala province or the northern Mae Hong Son province during 2007. In Yala, the parasites had identical flanking and neutral microsatellite haplotypes, which is consistent with our previously published results [26], indicating a closely related clonal population.

Population differentiation analysis further reveals that parasites with the C580Y allele group together by geography (Fig 3A). Analysis of raw microsatellite haplotype data for these parasites revealed that alleles circulating in the east and west comprise two distinct lineages marked by differences in the 8.6kb locus downstream of the K13 gene (Fig 2). These data suggest the C580Y mutations may have arisen independently along the Thai-Cambodia and Thai-Myanmar borders. The reduced pattern of heterozygosity of C580Y alleles compared to wild type alleles (Fig 3) further suggests recent independent origins along the Thai-Cambodia and Thai-Myanmar borders. This interpretation of our data is consistent with the recently published work by Miotto *et al*. [21]. The study provided compelling evidence for the selection of the C580Y allele in the Greater Mekong Subregion [21], which is consistent with our data, and suggested that the selection process may have been under way on both sides of Thailand at the time of this study. One possible interpretation of our findings would be that the parasites migrated across Thailand prior to the independent C580Y emergence events. Recent findings by Takala-Harrison *et al*. and Miotto *et al*. are consistent with the independent emergence of the C580Y allele, which was also observed along the Myanmar-Thai border and the lower Mekong region [13, 21]. The strong association between the genetic dissimilarity and geographic distance of the C580Y mutants further supports the hypothesis that this mutation may have emerged independently in eastern and western Thailand (Fig 5).

Given the history of population movements within this region, some of the mutant alleles in the Thai-Myanmar region (C580Y and P574L) and Thai-Cambodia region (C580Y and R539T) may share common ancestry. Interestingly, in the work by Miotto *et al*. [21], parasites with the most common K13 mutant alleles (C580Y, I543T, R539T, and Y493H) were found in multiple countries in the region, indicating that parasite cross-border movement may have already occurred. It remains to be determined if the P574L and R575K alleles, which have been found in Myanmar [13, 23], originated in either Thailand or Myanmar.

It has been suggested that in the absence of drug pressure, parasites with some resistant mutations are less fit than their ancestral wild type counterparts [27]. However, with continued drug pressure one might expect resistant alleles, such as the C580Y mutation, to eventually become fixed in the population as has occurred with other resistance mutations in the past. The work by Ariey *et al*., which identified the K13 propeller as a molecular marker of artemisinin resistance, demonstrated that over the course of 11 years (2001–2012), the C580Y allele prevalence increased from 40% to 90% in Pailin, Cambodia [19]. This would suggest that the parasites carrying the C580Y mutation may be nearing fixation in the population, and therefore, no sensitive parasites will remain to outcompete them in the absence of ACT. This is very worrisome, as ACT is one of our last working treatment options for drug resistant *P*. *falciparum*.

In summary, it is evident from our study that artemisinin-resistant K13 alleles have been evolving along both the Thai-Cambodian border and Thai-Myanmar border long before the artemisinin containment project was implemented. It is further evident that the most commonly found C580Y allele had two distinct haplotypes, suggesting different patterns of origin and migration along the Thai-Cambodia and Thai-Myanmar regions.

## Materials and Methods

### Ethics statement

This protocol was approved by the Thailand Ministry of Public Health. CDC Human Research Protection Office provided approval for retrospective testing using anonymized samples. Study participants and/or their guardians provided written informed consent.

### Study sites and samples

A total of 417 *Plasmodium falciparum* infected blood samples were used in this study. The samples were collected in 2007 as part of a malaria surveillance study conducted by the Thailand Ministry of Public Health [28]. Finger prick blood samples were collected from ten malaria-endemic provinces of Thailand. Six of these provinces (Mae Hong Son, Tak, Kanchanaburi, Prachuap, Chumporn, and Ranong) are on the Myanmar border, and three (Sisaket, Chanthaburi, and Trat) are on the Cambodian border, while one (Yala) is in southern Thailand bordering Malaysia. In 2007, the reported malaria incidence rates were 17.2, 9.2, 8.7, and 8.5 cases per 1,000 residents in Yala, Mae Hong Son, Tak, and Ranong, respectively, and 3.9, 2.9, 1.7 and 1.2 per 1,000 population in Chumporn, Prachuap, Chanthaburi and Kanchanaburi, respectively [29].

### PCR amplification and sequencing of *Plasmodium falciparum* K13 gene

The K13 gene was amplified using a nested PCR method that was modified from a previous study [19]. New secondary primers that are species specific for *P*. *falciparum* were developed and used. For the primary PCR the same primers as in [19] were used (K13P1 5’-GGGAATCTGGTGGTAACAGC-3’ and K13R1 5’-CGGAGTGACCAAATCTGGGA-3’). For the secondary PCR, new primers were designed (K13S1, 5’ GTAAAGTGAAGCCTTGTTG-3’ and K13S2 5'-TTCATTTGTATCTGGTGAAAAG -3’). Two μl of genomic DNA was amplified using 0.5 μM of each primer, 0.2 mM dNTP, 3 and 2 mM MgCl_2_ for the primary and secondary reactions, respectively, and 1 U Expand High Fidelity Taq (Roche). For the primary reaction, the following cycling parameters were used: 5 min at 94°C, 40 cycles of 94°C for 30 s, 60°C for 90s, 72°C for 90s, and final extension for 10 min at 72°C. For the nested PCR, 1 μl of the primary PCR product was used as a template. For the nested PCR reaction, the following cycling parameters were used: 2 min at 94°C, 40 cycles of 94°C for 30 s, 55°C for 30s, 72°C for 90s, and final extension for 10 min at 72°C. PCR products were separated and visualized using 2% agarose gel electrophoresis and Gel red (Biotium, Hayward CA). Sanger sequencing of PCR products was performed using ABI 3730 (Applied Biosystems, Foster City, CA). Sequences were deposited to Genbank (Accession Numbers:KP334284—KP334700).

### Microsatellite loci genotyping

Twenty-five microsatellite loci flanking the K13 gene on chromosome 13 (*PF3D7_1343700*) were tested for evidence of selection as indicated by a reduction in heterozygosity around the K13 gene. Three of the loci, L4_165 (72.3 kb), LM_173 (-3.74 kb) and B1_P1 (-31.9 kb) were previously described [18], and the remaining 22 were newly designed for this study. Only 12 out of 25 loci were informative and further analyzed to study the selective sweeps and genetic lineages of resistance K13 alleles (downstream: 3.4kb, 8.6kb, 15.1kb, 31.0kb, 31.5kb, 72.3kb; upstream: -0.15kb, -3.7kb, -6.36kb, -31.9kb, -50.0kb, -56.0kb). The primers used are shown in S1 Table. In addition, eight putatively neutral microsatellite loci located on chromosome 2 (GenBank UniSTS C2M27, C2M29, C2M34, and C2M33) and chromosome 3 (GenBank UniSTS C3M40, C3M88, C3M69, and C3M39) were used as previously described [28]. A previously described protocol [30] for cycling was modified for this study. Briefly, primer pairs with annealing temperature in the 50–60°C range were designed and cycling conditions were adjusted according to each primer pairs melting temperatures (TMs). The sizes of the amplification products were assayed by capillary electrophoresis on an Applied Biosystems 3130 xl sequencer (Applied Biosystems, Foster City CA).

### Estimating genetic diversity

To determine genetic diversity, the expected heterozygosity (H*e*) was estimated using all K13 flanking or neutral microsatellite loci using the Excel Microsatellite Toolkit, version 3.1.1 [31]. H*e* was calculated using the formula [*n*/ (*n*-1)][1-Ʃ*p*_*i*_^2^] for H*e*; and 2(*n*-1)/*n* ^3^ {2(*n*-2) [Ʃ(*p*_*i*_^3^-(Ʃ*p*_*i*_^2^)^2^]}for variance, where *n* is the number of samples genotyped for any locus and *p*_*i*_ is the frequency of the *i*th allele. Any locus for which an allele could not be amplified after two attempts was assigned as DNW, indicating no amplification. Mean H*e* between the wild type and C580Y mutant alleles were compared using the Mann-Whitney U test. Statistical significance was defined as p ≤ 0.05.

### Microsatellite and SNP analysis

Sanger sequences were analyzed using Geneious Pro R8 (www.geneious.com) to identify specific SNP combinations. A custom pipeline was created using the Geneious workflow feature to automate the SNP analysis (shared @GitHub). Briefly, by selecting a user defined sequence list (select all raw sequences > create list) and reference sequence as an input, the workflow will automatically map the input sequences to the reference sequence, identify all SNPs, and export the final SNP calls. Each step creates a sub-folder allowing the user to check the results. SNPs were only called if both the forward and reverse strands had the mutation. Microsatellite fragment analysis was performed using the Geneious Pro R8 microsatellite plugin. For determining genetic lineages of the K13 alleles, the POLYSAT R package was used [32]. Using the built-in functions in POLYSAT R, a pairwise distance matrix and principal component analysis (PCA) matrix was calculated using a stepwise mutation model for the flanking and neutral microsatellite markers. The pairwise distance matrix was used to construct a neighbor joining tree via the T-REX web server [33]. The resulting neighbor joining tree was imported into Geneious Pro R8 and colored according to geography. The geographic distance between the ten sampling sites was calculated, and genetic dissimilarity between each pair of isolates using the flanking microsatellites was plotted against the geographic distance between the sites where they were collected. Pearson’s correlation coefficient was calculated to assess the association between genetic dissimilarity and geographic distance. The genetic dissimilarity scatter plots were created and visualized using R 3.1.1. All R code used to run the analysis has been uploaded to: GitHub

### Accession numbers

Sequences were deposited to Genbank (Accession Numbers:KP334284—KP334700).

## Supporting Information

S1 Dataset The table comprises data from 417 Thai parasite isolates. Single nucleotide polymorphisms are color coded: S621F (blue), C580Y (red), R575K (orange), P574L (green), E556D (turquoise), R539T (purple), and N458Y (light blue). Allele lengths for flanking K13 are shown for twelve microsatellites positioned at: 72.3kb, 31.5kb, 31.0kb, 15.1kb, 8.6kb, 3.4kb, -0.15kb, -3.74kb, -6.36kb, -31.9kb, -50.0kb, and -56.0kb. In addition, allele lengths for neutral microsatellites positioned at: Chr3_335kb, Chr3_363kb, Chr3_383kb, Chr3_429kb, Chr2_302kb, Chr2_313kb, Chr2_319, and Chr2_380 are shown and color coded by Thai province. Teal green shading and lines indicate identical allele sizes. DNW (in grey) = indicates no successful amplification after a third attempt or not enough DNA was available to repeat the analysis.
(PDF)

S1 Fig Neighbor joining tree depicting relationship between K13 C580Y microsatellite haplotypes. Relationships among parasites with the C580Y allele, based on K13 flanking microsatellites (A) and neutral microsatellites (B). Provinces are abbreviated as follows: in the east, Chanthaburi (CB), Trat (TR), and Sisaket (SS); in the west, Tak (TK), Kanchanaburi (KN), Chumporn (CP), Ranong (RN). Red branches indicate parasite isolates from eastern Thailand (CB, TR, SS) and blue branches, parasites from western Thailand (TK, KN, CP, and RN).
(TIF)

S1 Table Table with microsatellite primers tested. (PDF)
