# Correction: Selection and Spread of Artemisinin-Resistant Alleles in Thailand Prior to the Global Artemisinin Resistance Containment Campaign

**DOI:** 10.1371/journal.ppat.1004862

**Published:** 2015-04-22

**Authors:** Eldin Talundzic, Sheila Akinyi Okoth, Kanungnit Congpuong, Mateusz M. Plucinski, Lindsay Morton, Ira F. Goldman, Patrick S. Kachur, Chansuda Wongsrichanalai, Wichai Satimai, John W. Barnwell, Venkatachalam Udhayakumar

The Funding Statement is missing an acknowledgment to the Advanced Molecular Detection Initiative at the CDC. The correct Funding Statement is provided here:

ET is supported by an American Society for Microbiology (ASM) and Centers for Disease Control and Prevention Postdoctoral Fellowship. We acknowledge support from the Advanced Molecular Detection Initiative at the CDC, the Atlanta Research and Education Foundation, Atlanta, GA, and the CDC Antimicrobial Resistance Working Group. The funders had no role in study design, data collection and analysis, decision to publish, or preparation of the manuscript.
