# Supplementary material for: Selection and Spread of Artemisinin-Resistant Alleles in Thailand Prior to the Global Artemisinin Resistance Containment Campaign
Source: PLoS Pathog. 2015 Apr 2;11(4):e1004789. doi: 10.1371/journal.ppat.1004789 (PMC4383523; doi:10.1371/journal.ppat.1004789)
Supplement: S1 Dataset — Single nucleotide polymorphisms are color coded: S621F (blue), C580Y (red), R575K (orange), P574L (green), E556D (turquoise), R539T (purple), and N458Y (light blue). Allele lengths for flanking K13 are shown for twelve microsatellites positioned at: 72.3kb, 31.5kb, 31.0kb, 15.1kb, 8.6kb, 3.4kb, -0.15kb, -3.74kb, -6.36kb, -31.9kb, -50.0kb, and -56.0kb. In addition, allele lengths for neutral microsatellites positioned at: Chr3_335kb, Chr3_363kb, Chr3_383kb, Chr3_429kb, Chr2_302kb, Chr2_313kb, Chr2_319, and Chr2_380 are shown and color coded by Thai province. Teal green shading and lines indicate identical allele sizes. DNW (in grey) = indicates no successful amplification after a third attempt or not enough DNA was available to repeat the analysis. (PDF) [file ppat.1004789.s001.pdf]

## SNPs / Flanking Microsatellites (K13)

| Group | Sample | N458Y | R539T | E556D | P574L | R575K | C580Y | S621F | REGION | 72.3kb | 31.5kb | 31.0kb | 15.1kb | 8.6kb | 3.4kb | -0.15kb | -3.74kb | -6.36kb | -31.9kb | -50.0kb | -56.0kb |
|---|---|---|---|---|---|---|---|---|---|---|---|---|---|---|---|---|---|---|---|---|---|
| | | N | R | E | P | R | C | S | EAST | 248 | 194 | 310 | 138 | 288 | 131 | 193 | 148 | 282 | 201 | 258 | 203 |
| C580Y | 1 | - | - | - | - | - | Y | - | EAST | - | - | 300 | - | - | - | - | - | - | - | - | 205 |
| | 2 | - | - | - | - | - | Y | - | EAST | DNW | - | - | - | DNW | - | - | - | - | DNW | - | - |
| | 3 | - | - | - | - | - | Y | - | EAST | - | - | - | - | - | - | - | - | - | - | - | 205 |
| | 4 | - | - | - | - | - | Y | - | EAST | DNW | - | - | - | - | - | - | - | - | DNW | DNW | DNW |
| | 5 | - | - | - | - | - | Y | - | EAST | 236 | - | - | - | - | - | - | - | - | DNW | - | - |
| | 6 | - | - | - | - | - | Y | - | EAST | DNW | - | - | - | DNW | - | - | - | - | DNW | - | - |
| | 7 | - | - | - | - | - | Y | - | EAST | 236 | - | - | - | - | - | - | - | - | - | - | - |
| | 8 | - | - | - | - | - | Y | - | EAST | 236 | - | 318 | - | - | - | - | - | - | - | - | - |
| | 9 | - | - | - | - | - | Y | - | EAST | DNW | - | - | - | DNW | - | - | - | - | - | - | - |
| | 10 | - | - | - | - | - | Y | - | EAST | 236 | - | 300 | - | - | - | - | - | - | - | - | - |
| | 11 | - | - | - | - | - | Y | - | EAST | 236 | - | - | - | - | - | - | - | - | - | - | DNW |
| | 12 | - | - | - | - | - | Y | - | EAST | DNW | - | 300 | - | DNW | DNW | - | - | - | - | - | - |
| | 13 | - | - | - | - | - | Y | - | EAST | DNW | - | 307 | - | DNW | - | - | - | DNW | - | - | - |
| | 14 | - | - | - | - | - | Y | - | EAST | DNW | - | 318 | - | - | - | - | - | - | - | - | - |
| | 15 | - | - | - | - | - | Y | - | EAST | DNW | DNW | DNW | - | - | - | - | - | - | 147 | DNW | DNW |
| | 16 | - | - | - | - | - | Y | - | EAST | DNW | DNW | DNW | - | - | - | - | - | - | - | DNW | DNW |
| | 17 | - | - | - | - | - | Y | - | WEST | DNW | - | - | - | 278 | - | - | 150 | - | DNW | DNW | - |
| | 18 | - | - | - | - | - | Y | - | WEST | 244 | 198 | - | - | 278 | 117 | - | 150 | - | - | - | - |
| | 19 | - | - | - | - | - | Y | - | WEST | DNW | - | 300 | - | DNW | - | - | - | - | DNW | - | - |
| | 20 | - | - | - | - | - | Y | - | WEST | DNW | 198 | - | - | DNW | DNW | - | - | - | - | - | - |
| | 21 | - | - | - | - | - | Y | - | WEST | - | 198 | - | - | 278 | - | - | - | - | - | - | - |
| | 22 | - | - | - | - | - | Y | - | WEST | - | 198 | - | - | 278 | - | - | - | - | - | - | - |
| | 23 | - | - | - | - | - | Y | - | WEST | DNW | - | - | - | 278 | - | - | - | 284 | DNW | - | - |
| | 24 | - | - | - | - | - | Y | - | WEST | DNW | 198 | - | - | 278 | - | - | - | - | 150 | - | 205 |
| | 25 | - | - | - | - | - | Y | - | WEST | DNW | 198 | - | - | 278 | - | - | - | DNW | - | DNW | - |
| | 26 | - | - | - | - | - | Y | - | WEST | DNW | 198 | - | - | 278 | DNW | - | - | - | DNW | - | - |
| R575K | 27 | - | - | - | - | K | - | - | WEST | DNW | - | 300 | - | DNW | 117 | 197 | 150 | 280 | DNW | - | - |
| | 28 | - | - | - | - | K | - | - | WEST | DNW | DNW | - | - | 195 | DNW | - | 150 | DNW | DNW | DNW | DNW |
| | 29 | - | - | - | - | K | - | - | WEST | DNW | - | 300 | - | 143 | DNW | - | 156 | - | DNW | - | - |
| | 30 | - | - | - | - | K | - | - | WEST | DNW | - | 300 | - | DNW | DNW | - | 150 | DNW | DNW | - | - |
| | 31 | - | - | - | - | K | - | - | WEST | DNW | DNW | 300 | - | - | - | - | 150 | 280 | DNW | DNW | DNW |
| | 32 | - | - | - | - | K | - | - | WEST | DNW | - | DNW | - | DNW | DNW | - | - | 266 | 124 | - | 205 |
| | 33 | - | - | - | - | K | - | - | WEST | DNW | 198 | - | - | DNW | DNW | - | - | - | 106 | - | - |
| | 34 | - | - | - | - | K | - | - | WEST | DNW | - | 300 | - | - | DNW | - | 150 | - | DNW | - | - |
| | 35 | - | - | - | - | K | - | - | WEST | 244 | - | 300 | - | DNW | DNW | - | - | - | DNW | - | - |
| | 36 | - | - | - | - | K | - | - | WEST | DNW | - | 300 | - | DNW | DNW | - | - | DNW | DNW | DNW | - |
| P574L | 37 | - | - | - | L | - | - | - | WEST | 238 | 198 | - | - | - | - | - | - | - | 210 | - | - |
| | 38 | - | - | - | L | - | - | - | WEST | DNW | 198 | - | - | - | - | - | - | - | 210 | - | - |
| | 39 | - | - | - | L | - | - | - | WEST | DNW | 198 | - | - | - | - | - | - | - | 210 | - | - |
| | 40 | - | - | - | L | - | - | - | WEST | DNW | 198 | - | - | 215 | - | - | - | - | 210 | DNW | 205 |
| | 41 | - | - | - | L | - | - | - | WEST | DNW | 198 | 305 | - | - | - | - | - | - | 210 | - | 205 |
| | 42 | - | - | - | L | - | - | - | WEST | DNW | 198 | - | - | - | - | - | - | - | 106 | DNW | DNW |
| E556D | 43 | - | - | D | - | - | - | - | WEST | - | 204 | - | - | - | - | 197 | 150 | 274 | 204 | - | - |
| S621F | 44 | - | - | - | - | - | - | F | WEST | 238 | DNW | DNW | - | - | - | - | - | - | 210 | DNW | DNW |
| N458Y | 45 | Y | - | - | - | - | - | - | WEST | DNW | DNW | DNW | - | 278 | - | - | - | - | DNW | DNW | DNW |
| | 46 | Y | - | - | - | - | - | - | WEST | DNW | DNW | DNW | - | - | - | - | - | - | DNW | DNW | DNW |
| | 47 | Y | - | - | - | - | - | - | WEST | 238 | DNW | DNW | - | - | - | - | - | - | 210 | DNW | DNW |
| R539T | 48 | - | T | - | - | - | - | - | EAST | 236 | 204 | - | - | 266 | 123 | - | - | - | - | - | - |
| | 49 | - | T | - | - | - | - | - | EAST | DNW | DNW | - | - | 266 | 123 | - | - | - | DNW | DNW | DNW |
| | 50 | - | T | - | - | - | - | - | EAST | 236 | - | 307 | - | DNW | 123 | - | - | DNW | DNW | - | - |

## Neutral Microsatellites

| Area | 3_335 kb | 3_363 kb | 3_383 kb | 3_429 kb | 2_302 | 2_313 | 2_319 | 2_380 |
|---|---|---|---|---|---|---|---|---|
| | | | | | | | | |
| Chanthaburi | 126 | 152 | 138 | 135 | 190 | 230 | 109 | 111 |
| Chanthaburi | 124 | 1 | 140 | 134 | 195 | 228 | 113 | 1 |
| Chanthaburi | 122 | 143 | 124 | 122 | 184 | 234 | 108 | 111 |
| Chanthaburi | 122 | 143 | 124 | 122 | 184 | 234 | 108 | 111 |
| Sisaket | 133 | 141 | 147 | 134 | 184 | 238 | 107 | 109 |
| Sisaket | 1 | 141 | 147 | 134 | 184 | 238 | 107 | 1 |
| Sisaket | 133 | 141 | 147 | 134 | 184 | 238 | 107 | 111 |
| Sisaket | 133 | 141 | 147 | 134 | 183 | 238 | 107 | 111 |
| Sisaket | 133 | 141 | 147 | 134 | 184 | 238 | 107 | 111 |
| Sisaket | 133 | 141 | 147 | 134 | 184 | 238 | 107 | 111 |
| Sisaket | 122 | 135 | 124 | 134 | 184 | 223 | 111 | 111 |
| Trat | 138 | 141 | 122 | 134 | 194 | 234 | 111 | 1 |
| Trat | 138 | 141 | 122 | 119+134 | 1 | 226 | 1 | 1 |
| Trat | 138 | 141 | 122 | 134 | 190 | 226 | 107 | 1 |
| Trat | 138 | 141 | 122 | 135 | 190 | 226 | 107 | 111 |
| Trat | 138 | 141 | 122 | 134 | 190 | 226 | 107 | 91 |
| Chumphon | 116 | 146 | 122 | 135 | 181 | 263 | 111 | 103+111 |
| Chumphon | 122 | 153 | 124 | 134 | 191 | 236 | 111 | 78 |
| Kanchanaburi | 1 | 1 | 1 | 1 | 183 | 1 | 103 | 1 |
| Kanchanaburi | 126 | 153 | 158 | 119 | 185 | 232 | 117 | 110.79+118.99 |
| Kanchanaburi | 100 | 144 | 126 | 135 | 190 | 232 | 107 | 121 |
| Kanchanaburi | 118 | 141 | 158 | 120 | 179 | 1 | 105 | 127 |
| Kanchanaburi | 116 | 146 | 126 | 135 | 189 | 232 | 107 | 121 |
| Ranong | 122 | 135 | 132 | 1 | 185 | 234 | 107 | 78 |
| Ranong | 122 | 146 | 148 | 135 | 182 | 263 | 101 | 111 |
| Tak | 122 | 152 | 125 | 150 | 189 | 232 | 107 | 121.02+96.97 |
| Kanchanaburi | 114 | 1 | 1 | 135 | 1 | 1 | 1 | 1 |
| Kanchanaburi | 126 | 1 | 1 | 1 | 1 | 1 | 107 | 1 |
| Kanchanaburi | 122 | 134 | 124 | 135 | 1 | 234 | 107 | 78 |
| Kanchanaburi | 120 | 1 | 124 | 1 | 1 | 1 | 106 | 1 |
| Prachuap | 114 | 146 | 124 | 120 | 1 | 1 | 113 | 105 |
| Prachuap | 114 | 148 | 124 | 135 | 186 | 1 | 113 | 78+105 |
| Prachuap | 100 | 1 | 1 | 1 | 1 | 1 | 1 | 1 |
| Prachuap | 114 | 1 | 1 | 135 | 1 | 1 | 1 | 1 |
| Prachuap | 114 | 148 | 1 | 135 | 179 | 1 | 113 | 105 |
| Prachuap | 1 | 153 | 1 | 1 | 1 | 1 | 1 | 1 |
| Chumphon | 125 | 148 | 122 | 135 | 186 | 231 | 117 | 111 |
| Prachuap | 125 | 148 | 124 | 151 | 181 | 263 | 111 | 115 |
| Ranong | 128 | 141 | 158 | 135 | 195 | 241 | 103 | 78 |
| Ranong | 125 | 135+153 | 122 | 135 | 179 | 233 | 113 | 78+110 |
| Ranong | 125 | 155 | 148 | 135 | 187 | 236 | 115 | 78 |
| Ranong | 125 | 155 | 124 | 135 | 197 | 247 | 109 | 105 |
| Chumphon | 151 | 153 | 125 | 151 | 180 | 233 | 113 | 113 |
| Tak | 130 | 1 | 124 | 1 | 199 | 1 | 107 | 78 |
| Kanchanaburi | 116 | 146 | 125 | 135 | 189 | 232 | 107 | 121 |
| Tak | 116 | 152 | 162 | 134 | 187 | 234 | 98 | 95 |
| Tak | 116 | 152 | 162 | 134 | 183 | 235+247 | 105 | 95 |
| Sisaket | 120 | 141 | 121 | 139 | 186 | 245 | 119 | 101 |
| Sisaket | 127 | 135 | 158 | 134 | 184 | 236 | 107 | 94 |
| Trat | 122 | 146 | 121 | 134 | 189 | 230 | 107 | 76 |
| 3D7 | 122.86 | 145.85 | 172.01 | 156.76 | 193.28 | 262.4 | 138.89 | 115.09 |
| 3D7 | 123.02 | 145.73 | 173.17 | 156.76 | 193.33 | 262.4 | 137.37 | 115.02 |
| HB3 | | | | | | | | |
| HB3 | | | | | | | | |
| 7G8 | 119.56 | 147.79 | 151.38 | 150.69 | | | | |
| 7G8 | 120.17 | 147.67 | 151.17 | 150.7 | | | | |
| K1 | | | | | | | | |
| K1 | | | | | | | | |
| | 123.6 | 151.43 | 146.68 | 139.11 | 191.3 | 225.63 | 127.21 | 116.79 |
| | 124.1 | 151.45 | 147.37 | 137.83 | 191.27 | 225.41 | 127.21 | 116.88 |

## Controls (Lab isolates)

| | 72.3kb | 31.5kb | 31.0kb | 15.1kb | 8.6kb | 3.4kb | -0.15kb | -3.74kb | -6.36kb | -31.9kb | -50.0kb | -56.0kb |
|---|---|---|---|---|---|---|---|---|---|---|---|---|
| 3D7 | 242 | 198 | 300 | 147 | 274 | DNW | 225 | 164 | - | 207 | 264 | 218 |
| 7G8 | | 207 | - | 144 | 261 | 102 | - | 156 | 280 | - | 260 | 220 |
| D6 | | - | 282 | 147 | 282 | 126 | 126 | 160 | 272 | - | - | 206 |
| FCR3 | | - | - | - | - | - | - | - | - | - | - | |
| HB3 | - | 188 | 300 | 140 | 278 | 141 | 206 | 150 | 274 | - | 272 | |
| W2 | 244 | 198 | - | - | 278 | - | - | - | - | 201 | - | |

## SNPs (All wild type) / Flanking Microsatellites (K13)

| Sample | N458Y | R539T | E556D | P574L | R575K | C580Y | S621F | REGION | 72.3kb | 31.5kb | 31.0kb | 15.1kb | 8.6kb | 3.4kb | -0.15kb | 3.7kb | -6.36kb | -31.9kb | -50.0kb | -56.0kb |
|---|---|---|---|---|---|---|---|---|---|---|---|---|---|---|---|---|---|---|---|---|
| | N | R | E | P | R | C | S | EAST | 248 | 194 | 310 | 138 | 288 | 131 | 193 | 148 | 282 | 201 | 258 | 203 |
| 51 | - | - | - | - | - | - | - | EAST | 232 | - | - | - | DNW | - | - | - | DNW | DNW | - | - |
| 52 | - | - | - | - | - | - | - | EAST | 232 | DNW | DNW | - | - | - | - | 150 | - | DNW | DNW | DNW |
| 53 | - | - | - | - | - | - | - | EAST | 248 | - | - | 144 | DNW | 121 | 197 | 150 | 268 | DNW | - | 205 |
| 54 | - | - | - | - | - | - | - | EAST | 244 | - | 305 | - | 278 | - | - | - | - | DNW | - | 205 |
| 55 | - | - | - | - | - | - | - | EAST | DNW | 198 | - | 144 | 280 | 121 | 197 | 150 | 268 | - | DNW | DNW |
| 56 | - | - | - | - | - | - | - | WEST | 238 | 204 | - | - | - | - | 197 | 150 | 274 | 204 | - | 205 |
| 57 | - | - | - | - | - | - | - | WEST | DNW | 198 | - | - | - | 119 | - | - | 266 | - | - | - |
| 58 | - | - | - | - | - | - | - | WEST | DNW | - | 300 | - | 278 | - | - | - | - | - | - | - |
| 59 | - | - | - | - | - | - | - | WEST | DNW | 198 | - | - | DNW | - | - | - | DNW | DNW | - | - |
| 60 | - | - | - | - | - | - | - | WEST | DNW | 198 | 300 | - | - | 115 | - | - | - | 218 | - | - |
| 61 | - | - | - | - | - | - | - | WEST | 238 | DNW | DNW | - | - | 129 | - | - | 266 | DNW | - | DNW |
| 62 | - | - | - | - | - | - | - | WEST | DNW | 204 | 300 | - | - | - | 197 | 150 | 274 | - | - | - |
| 63 | - | - | - | - | - | - | - | WEST | - | DNW | DNW | - | 278 | - | - | - | - | 145 | - | DNW |
| 64 | - | - | - | - | - | - | - | WEST | DNW | DNW | DNW | - | 278 | 119 | 197 | 150 | 274 | DNW | DNW | DNW |
| 65 | - | - | - | - | - | - | - | WEST | DNW | 198 | DNW | - | DNW | - | - | - | DNW | - | - | - |
| 66 | - | - | - | - | - | - | - | WEST | - | DNW | - | - | 278 | - | - | - | - | - | DNW | DNW |
| 67 | - | - | - | - | - | - | - | WEST | 244 | DNW | - | - | 273 | - | DNW | 150 | DNW | DNW | DNW | DNW |
| 68 | - | - | - | - | - | - | - | WEST | DNW | DNW | DNW | - | - | - | 197 | 150 | 274 | 204 | - | DNW |
| 69 | - | - | - | - | - | - | - | WEST | 244 | DNW | DNW | - | - | - | - | - | - | DNW | DNW | DNW |
| 70 | - | - | - | - | - | - | - | WEST | DNW | DNW | DNW | - | 278 | - | - | - | - | - | - | DNW |
| 71 | - | - | - | - | - | - | - | WEST | - | 190 | - | - | DNW | - | - | - | - | DNW | DNW | DNW |
| 72 | - | - | - | - | - | - | - | WEST | 238 | - | - | - | DNW | - | - | - | - | - | - | - |
| 73 | - | - | - | - | - | - | - | WEST | 238 | - | 300 | - | 278 | - | - | - | DNW | 141 | - | - |
| 74 | - | - | - | - | - | - | - | WEST | 240 | 198 | 300 | - | - | - | - | - | - | - | DNW | DNW |
| 75 | - | - | - | - | - | - | - | WEST | DNW | 202 | 295 | - | - | - | - | - | - | - | DNW | DNW |
| 76 | - | - | - | - | - | - | - | WEST | 242 | 198 | 300 | - | - | 115 | - | - | - | - | - | - |
| 77 | - | - | - | - | - | - | - | WEST | DNW | - | 300 | - | 278 | 119 | 197 | 150 | 274 | 204 | - | - |
| 78 | - | - | - | - | - | - | - | WEST | DNW | - | - | - | DNW | 115 | - | - | DNW | - | - | - |
| 79 | - | - | - | - | - | - | - | WEST | DNW | - | 300 | - | - | - | - | 150 | 280 | 207 | - | - |
| 80 | - | - | - | - | - | - | - | WEST | 246 | DNW | DNW | - | - | - | - | 150 | 280 | DNW | DNW | DNW |
| 81 | - | - | - | - | - | - | - | WEST | 244 | 198 | - | - | 278 | - | - | - | - | 205 | - | - |
| 82 | - | - | - | - | - | - | - | WEST | - | DNW | 300 | - | - | - | - | - | - | 211 | DNW | DNW |
| 83 | - | - | - | - | - | - | - | WEST | DNW | - | DNW | - | 278 | - | - | - | - | - | - | - |
| 84 | - | - | - | - | - | - | - | WEST | - | 198 | - | 144 | DNW | 112 | - | 150 | 271 | DNW | - | - |
| 85 | - | - | - | - | - | - | - | WEST | 238 | - | - | - | DNW | - | - | - | - | - | - | - |
| 86 | - | - | - | - | - | - | - | WEST | 250 | 198 | DNW | - | DNW | 115 | - | - | - | 204 | DNW | DNW |
| 87 | - | - | - | - | - | - | - | WEST | DNW | 198 | DNW | - | - | 115 | - | - | - | DNW | - | - |
| 88 | - | - | - | - | - | - | - | WEST | 238 | 198 | - | - | - | 115 | - | - | - | 218 | - | - |
| 89 | - | - | - | - | - | - | - | WEST | 244 | 198 | - | - | - | - | - | - | - | 210 | - | - |
| 90 | - | - | - | - | - | - | - | WEST | 236 | 198 | 272 | - | 293 | 129 | - | - | 266 | 210 | - | - |
| 91 | - | - | - | - | - | - | - | WEST | DNW | - | - | - | 278 | - | - | - | - | 210 | - | 205 |
| 92 | - | - | - | - | - | - | - | WEST | DNW | - | - | 144 | DNW | 113 | - | - | 277 | 208 | - | - |
| 93 | - | - | - | - | - | - | - | WEST | 238 | 198 | - | 144 | 278 | - | - | - | - | 210 | - | 205 |
| 94 | - | - | - | - | - | - | - | WEST | DNW | 198 | DNW | - | DNW | - | - | - | - | - | - | 205 |
| 95 | - | - | - | - | - | - | - | WEST | 250 | - | - | - | 278 | - | - | - | - | - | - | 201 |
| 96 | - | - | - | - | - | - | - | WEST | 250 | - | - | - | 280 | 121 | 191 | DNW | DNW | 206 | DWN | - |
| 97 | - | - | - | - | - | - | - | EAST | 240 | - | - | - | DNW | - | - | - | - | - | - | - |
| 98 | - | - | - | - | - | - | - | EAST | DNW | 204 | 305 | - | 266 | 123 | - | - | - | - | - | - |
| 99 | - | - | - | - | - | - | - | EAST | 236 | - | - | - | 282 | 125 | 197 | 150 | 285 | 208 | - | - |
| 100 | - | - | - | - | - | - | - | EAST | DNW | 198 | - | - | - | - | 197 | 154 | 288 | 216 | - | 213 |
| 101 | - | - | - | - | - | - | - | WEST | DNW | 198 | - | - | DNW | 127 | - | 157 | DNW | DNW | DNW | 205 |
| 102 | - | - | - | - | - | - | - | WEST | DNW | DNW | DNW | - | - | 140 | 190 | - | 280 | - | DNW | DNW |
| 103 | - | - | - | - | - | - | - | WEST | DNW | 198 | DNW | - | - | 115 | - | - | DNW | DNW | DNW | 205 |
| 104 | - | - | - | - | - | - | - | WEST | DNW | DNW | DNW | - | 278 | 119 | 197 | 150 | 274 | 209 | DNW | DNW |
| 105 | - | - | - | - | - | - | - | WEST | DNW | 198 | 300 | - | - | 115 | - | - | - | 218 | - | - |
| 106 | - | - | - | - | - | - | - | WEST | DNW | - | - | - | DNW | - | - | - | DNW | - | - | - |
| 107 | - | - | - | - | - | - | - | WEST | DNW | DNW | DNW | - | 266 | 123 | - | 150 | 274 | DNW | DNW | DNW |
| 108 | - | - | - | - | - | - | - | WEST | DNW | DNW | DNW | - | DNW | 129 | - | 144 | 268 | DNW | DNW | DNW |
| 109 | - | - | - | - | - | - | - | WEST | DNW | DNW | DNW | - | - | - | - | 150 | 280 | DNW | DNW | DNW |
| 110 | - | - | - | - | - | - | - | WEST | DNW | DNW | DNW | - | 297 | 127 | - | 155 | 277 | DNW | DNW | DNW |
| 111 | - | - | - | - | - | - | - | WEST | DNW | DNW | DNW | 141 | 284 | 127 | 197 | 153 | 280 | DNW | DNW | DNW |
| 112 | - | - | - | - | - | - | - | WEST | 236 | DNW | DNW | - | 278 | - | - | - | - | - | DNW | DNW |
| 113 | - | - | - | - | - | - | - | WEST | DNW | 202 | - | - | DNW | - | 197 | 150 | DNW | 135 | - | - |
| 114 | - | - | - | - | - | - | - | EAST | - | - | - | - | - | - | - | - | - | - | - | - |
| 115 | - | - | - | - | - | - | - | EAST | DNW | - | - | - | DNW | - | - | - | DNW | - | - | 205 |
| 116 | - | - | - | - | - | - | - | EAST | DNW | DNW | - | 144 | 280 | 121 | 197 | 150 | 268 | DNW | DNW | DNW |
| 117 | - | - | - | - | - | - | - | EAST | DNW | - | 305 | - | - | - | - | - | - | DNW | - | - |
| 118 | - | - | - | - | - | - | - | WEST | 236 | 198 | - | - | - | 119 | 197 | 150 | 277 | 141 | - | 205 |
| 119 | - | - | - | - | - | - | - | WEST | - | 198 | - | - | DNW | 119 | 197 | 150 | 277 | DNW | - | 205 |
| 120 | - | - | - | - | - | - | - | WEST | 250 | 198 | - | - | DNW | DNW | 197 | 150 | 277 | DNW | - | 205 |
| 121 | - | - | - | - | - | - | - | WEST | DNW | 198 | - | - | DNW | 119 | 197 | 150 | 277 | DNW | - | 205 |
| 122 | - | - | - | - | - | - | - | WEST | 240 | 198 | - | - | DNW | DNW | 197 | 150 | 277 | DNW | DNW | DNW |

| Sample | N458Y | R539T | E556D | P574L | R575K | C580Y | S621F |
|---|---|---|---|---|---|---|---|
| | - | - | - | - | - | - | - |
| 123 | - | - | - | - | - | - | - |
| 124 | - | - | - | - | - | - | - |
| 125 | - | - | - | - | - | - | - |
| 126 | - | - | - | - | - | - | - |
| 127 | - | - | - | - | - | - | - |
| 128 | - | - | - | - | - | - | - |
| 129 | - | - | - | - | - | - | - |
| 130 | - | - | - | - | - | - | - |
| 131 | - | - | - | - | - | - | - |
| 132 | - | - | - | - | - | - | - |
| 133 | - | - | - | - | - | - | - |
| 134 | - | - | - | - | - | - | - |
| 135 | - | - | - | - | - | - | - |
| 136 | - | - | - | - | - | - | - |
| 137 | - | - | - | - | - | - | - |
| 138 | - | - | - | - | - | - | - |
| 139 | - | - | - | - | - | - | - |
| 140 | - | - | - | - | - | - | - |
| 141 | - | - | - | - | - | - | - |
| 142 | - | - | - | - | - | - | - |
| 143 | - | - | - | - | - | - | - |
| 144 | - | - | - | - | - | - | - |
| 145 | - | - | - | - | - | - | - |
| 146 | - | - | - | - | - | - | - |
| 147 | - | - | - | - | - | - | - |
| 148 | - | - | - | - | - | - | - |
| 149 | - | - | - | - | - | - | - |
| 150 | - | - | - | - | - | - | - |
| 151 | - | - | - | - | - | - | - |
| 152 | - | - | - | - | - | - | - |
| 153 | - | - | - | - | - | - | - |
| 154 | - | - | - | - | - | - | - |
| 155 | - | - | - | - | - | - | - |
| 156 | - | - | - | - | - | - | - |
| 157 | - | - | - | - | - | - | - |
| 158 | - | - | - | - | - | - | - |
| 159 | - | - | - | - | - | - | - |
| 160 | - | - | - | - | - | - | - |
| 161 | - | - | - | - | - | - | - |
| 162 | - | - | - | - | - | - | - |
| 163 | - | - | - | - | - | - | - |
| 164 | - | - | - | - | - | - | - |
| 165 | - | - | - | - | - | - | - |
| 166 | - | - | - | - | - | - | - |
| 167 | - | - | - | - | - | - | - |
| 168 | - | - | - | - | - | - | - |
| 169 | - | - | - | - | - | - | - |
| 170 | - | - | - | - | - | - | - |
| 171 | - | - | - | - | - | - | - |
| 172 | - | - | - | - | - | - | - |
| 173 | - | - | - | - | - | - | - |
| 174 | - | - | - | - | - | - | - |
| 175 | - | - | - | - | - | - | - |
| 176 | - | - | - | - | - | - | - |
| 177 | - | - | - | - | - | - | - |
| 178 | - | - | - | - | - | - | - |
| 179 | - | - | - | - | - | - | - |
| 180 | - | - | - | - | - | - | - |
| 181 | - | - | - | - | - | - | - |
| 182 | - | - | - | - | - | - | - |
| 183 | - | - | - | - | - | - | - |
| 184 | - | - | - | - | - | - | - |
| 185 | - | - | - | - | - | - | - |
| 186 | - | - | - | - | - | - | - |
| 187 | - | - | - | - | - | - | - |
| 188 | - | - | - | - | - | - | - |
| 189 | - | - | - | - | - | - | - |
| 190 | - | - | - | - | - | - | - |
| 191 | - | - | - | - | - | - | - |
| 192 | - | - | - | - | - | - | - |
| 193 | - | - | - | - | - | - | - |
| 194 | - | - | - | - | - | - | - |
| 195 | - | - | - | - | - | - | - |
| 196 | - | - | - | - | - | - | - |
| 197 | - | - | - | - | - | - | - |
| 198 | - | - | - | - | - | - | - |
| 199 | - | - | - | - | - | - | - |
| 200 | - | - | - | - | - | - | - |
| 201 | - | - | - | - | - | - | - |
| 202 | - | - | - | - | - | - | - |
| 203 | - | - | - | - | - | - | - |
| 204 | - | - | - | - | - | - | - |
| 205 | - | - | - | - | - | - | - |
| 206 | - | - | - | - | - | - | - |
| 207 | - | - | - | - | - | - | - |
| 208 | - | - | - | - | - | - | - |
| 209 | - | - | - | - | - | - | - |
| 210 | - | - | - | - | - | - | - |
| 211 | - | - | - | - | - | - | - |
| 212 | - | - | - | - | - | - | - |
| 213 | - | - | - | - | - | - | - |
| 214 | - | - | - | - | - | - | - |
| 215 | - | - | - | - | - | - | - |
| 216 | - | - | - | - | - | - | - |
| 217 | - | - | - | - | - | - | - |
| 218 | - | - | - | - | - | - | - |
| 219 | - | - | - | - | - | - | - |
| 220 | - | - | - | - | - | - | - |
| 221 | - | - | - | - | - | - | - |
| 222 | - | - | - | - | - | - | - |
| 223 | - | - | - | - | - | - | - |
| 224 | - | - | - | - | - | - | - |
| 225 | - | - | - | - | - | - | - |
| 226 | - | - | - | - | - | - | - |
| 227 | - | - | - | - | - | - | - |
| 228 | - | - | - | - | - | - | - |
| 229 | - | - | - | - | - | - | - |
| 230 | - | - | - | - | - | - | - |
| 231 | - | - | - | - | - | - | - |
| 232 | - | - | - | - | - | - | - |
| 233 | - | - | - | - | - | - | - |
| 234 | - | - | - | - | - | - | - |
| 235 | - | - | - | - | - | - | - |
| 236 | - | - | - | - | - | - | - |
| 237 | - | - | - | - | - | - | - |
| 238 | - | - | - | - | - | - | - |
| 239 | - | - | - | - | - | - | - |
| 240 | - | - | - | - | - | - | - |
| 241 | - | - | - | - | - | - | - |
| 242 | - | - | - | - | - | - | - |
| 243 | - | - | - | - | - | - | - |
| 244 | - | - | - | - | - | - | - |
| 245 | - | - | - | - | - | - | - |
| 246 | - | - | - | - | - | - | - |
| 247 | - | - | - | - | - | - | - |
| 248 | - | - | - | - | - | - | - |
| 249 | - | - | - | - | - | - | - |
| 250 | - | - | - | - | - | - | - |
| 251 | - | - | - | - | - | - | - |
| 252 | - | - | - | - | - | - | - |
| 253 | - | - | - | - | - | - | - |
| 254 | - | - | - | - | - | - | - |
| 255 | - | - | - | - | - | - | - |
| 256 | - | - | - | - | - | - | - |
| 257 | - | - | - | - | - | - | - |
| 258 | - | - | - | - | - | - | - |
| 259 | - | - | - | - | - | - | - |
| 260 | - | - | - | - | - | - | - |
| 261 | - | - | - | - | - | - | - |
| 262 | - | - | - | - | - | - | - |
| 263 | - | - | - | - | - | - | - |
| 264 | - | - | - | - | - | - | - |
| 265 | - | - | - | - | - | - | - |
| 266 | - | - | - | - | - | - | - |
| 267 | - | - | - | - | - | - | - |
| 268 | - | - | - | - | - | - | - |
| 269 | - | - | - | - | - | - | - |
| 270 | - | - | - | - | - | - | - |
| 271 | - | - | - | - | - | - | - |
| 272 | - | - | - | - | - | - | - |
| 273 | - | - | - | - | - | - | - |
| 274 | - | - | - | - | - | - | - |
| 275 | - | - | - | - | - | - | - |
| 276 | - | - | - | - | - | - | - |
| 277 | - | - | - | - | - | - | - |
| 278 | - | - | - | - | - | - | - |
| 279 | - | - | - | - | - | - | - |
| 280 | - | - | - | - | - | - | - |
| 281 | - | - | - | - | - | - | - |
| 282 | - | - | - | - | - | - | - |
| 283 | - | - | - | - | - | - | - |
| 284 | - | - | - | - | - | - | - |
| 285 | - | - | - | - | - | - | - |
| 286 | - | - | - | - | - | - | - |
| 287 | - | - | - | - | - | - | - |
| 288 | - | - | - | - | - | - | - |
| 289 | - | - | - | - | - | - | - |
| 290 | - | - | - | - | - | - | - |
| 291 | - | - | - | - | - | - | - |
| 292 | - | - | - | - | - | - | - |
| 293 | - | - | - | - | - | - | - |
| 294 | - | - | - | - | - | - | - |
| 295 | - | - | - | - | - | - | - |
| 296 | - | - | - | - | - | - | - |
| 297 | - | - | - | - | - | - | - |
| 298 | - | - | - | - | - | - | - |
| 299 | - | - | - | - | - | - | - |
| 300 | - | - | - | - | - | - | - |
| 301 | - | - | - | - | - | - | - |
| 302 | - | - | - | - | - | - | - |
| 303 | - | - | - | - | - | - | - |
| 304 | - | - | - | - | - | - | - |
| 305 | - | - | - | - | - | - | - |
| 306 | - | - | - | - | - | - | - |
| 307 | - | - | - | - | - | - | - |
| 308 | - | - | - | - | - | - | - |
| 309 | - | - | - | - | - | - | - |
| 310 | - | - | - | - | - | - | - |
| 311 | - | - | - | - | - | - | - |
| 312 | - | - | - | - | - | - | - |
| 313 | - | - | - | - | - | - | - |
| 314 | - | - | - | - | - | - | - |
| 315 | - | - | - | - | - | - | - |
| 316 | - | - | - | - | - | - | - |
| 317 | - | - | - | - | - | - | - |
| 318 | - | - | - | - | - | - | - |
| 319 | - | - | - | - | - | - | - |
| 320 | - | - | - | - | - | - | - |
| 321 | - | - | - | - | - | - | - |
| 322 | - | - | - | - | - | - | - |
| 323 | - | - | - | - | - | - | - |
| 324 | - | - | - | - | - | - | - |
| 325 | - | - | - | - | - | - | - |
| 326 | - | - | - | - | - | - | - |
| 327 | - | - | - | - | - | - | - |
| 328 | - | - | - | - | - | - | - |
| 329 | - | - | - | - | - | - | - |
| 330 | - | - | - | - | - | - | - |
| 331 | - | - | - | - | - | - | - |
| 332 | - | - | - | - | - | - | - |
| 333 | - | - | - | - | - | - | - |
| 334 | - | - | - | - | - | - | - |
| 335 | - | - | - | - | - | - | - |
| 336 | - | - | - | - | - | - | - |
| 337 | - | - | - | - | - | - | - |
| 338 | - | - | - | - | - | - | - |
| 339 | - | - | - | - | - | - | - |
| 340 | - | - | - | - | - | - | - |
| 341 | - | - | - | - | - | - | - |
| 342 | - | - | - | - | - | - | - |
| 343 | - | - | - | - | - | - | - |
| 344 | - | - | - | - | - | - | - |
| 345 | - | - | - | - | - | - | - |
| 346 | - | - | - | - | - | - | - |
| 347 | - | - | - | - | - | - | - |
| 348 | - | - | - | - | - | - | - |
| 349 | - | - | - | - | - | - | - |
| 350 | - | - | - | - | - | - | - |
| 351 | - | - | - | - | - | - | - |
| 352 | - | - | - | - | - | - | - |
| 353 | - | - | - | - | - | - | - |
| 354 | - | - | - | - | - | - | - |
| 355 | - | - | - | - | - | - | - |
| 356 | - | - | - | - | - | - | - |
| 357 | - | - | - | - | - | - | - |
| 358 | - | - | - | - | - | - | - |
| 359 | - | - | - | - | - | - | - |
| 360 | - | - | - | - | - | - | - |
| 361 | - | - | - | - | - | - | - |
| 362 | - | - | - | - | - | - | - |
| 363 | - | - | - | - | - | - | - |
| 364 | - | - | - | - | - | - | - |
| 365 | - | - | - | - | - | - | - |
| 366 | - | - | - | - | - | - | - |
| 367 | - | - | - | - | - | - | - |
| 368 | - | - | - | - | - | - | - |
| 369 | - | - | - | - | - | - | - |
| 370 | - | - | - | - | - | - | - |
| 371 | - | - | - | - | - | - | - |
| 372 | - | - | - | - | - | - | - |
| 373 | - | - | - | - | - | - | - |
| 374 | - | - | - | - | - | - | - |
| 375 | - | - | - | - | - | - | - |
| 376 | - | - | - | - | - | - | - |
| 377 | - | - | - | - | - | - | - |
| 378 | - | - | - | - | - | - | - |
| 379 | - | - | - | - | - | - | - |
| 380 | - | - | - | - | - | - | - |
| 381 | - | - | - | - | - | - | - |
| 382 | - | - | - | - | - | - | - |
| 383 | - | - | - | - | - | - | - |
| 384 | - | - | - | - | - | - | - |
| 385 | - | - | - | - | - | - | - |
| 386 | - | - | - | - | - | - | - |
| 387 | - | - | - | - | - | - | - |
| 388 | - | - | - | - | - | - | - |
| 389 | - | - | - | - | - | - | - |
| 390 | - | - | - | - | - | - | - |
| 391 | - | - | - | - | - | - | - |
| 392 | - | - | - | - | - | - | - |
| 393 | - | - | - | - | - | - | - |
| 394 | - | - | - | - | - | - | - |
| 395 | - | - | - | - | - | - | - |
| 396 | - | - | - | - | - | - | - |
| 397 | - | - | - | - | - | - | - |
| 398 | - | - | - | - | - | - | - |
| 399 | - | - | - | - | - | - | - |
| 400 | - | - | - | - | - | - | - |
| 401 | - | - | - | - | - | - | - |
| 402 | - | - | - | - | - | - | - |
| 403 | - | - | - | - | - | - | - |
| 404 | - | - | - | - | - | - | - |
| 405 | - | - | - | - | - | - | - |
| 406 | - | - | - | - | - | - | - |
| 407 | - | - | - | - | - | - | - |
| 408 | - | - | - | - | - | - | - |
| 409 | - | - | - | - | - | - | - |
| 410 | - | - | - | - | - | - | - |
| 411 | - | - | - | - | - | - | - |
| 412 | - | - | - | - | - | - | - |
| 413 | - | - | - | - | - | - | - |
| 414 | - | - | - | - | - | - | - |
| 415 | - | - | - | - | - | - | - |
| 416 | - | - | - | - | - | - | - |
| 417 | - | - | - | - | - | - | - |

## FLANKING K13 MS (C580Y)

| Group | 72.3kb | 31.5kb | 31.0kb | 15.1kb | 8.6kb | 3.4kb | -0.15kb | -3.74kb | -6.36kb | -31.9kb | -50.0kb | -56.0kb |
|---|---|---|---|---|---|---|---|---|---|---|---|---|
| C580Y EASTERN THAILAND | 248 | 194 | 310 | 138 | 288 | 131 | 193 | 148 | 282 | 201 | 258 | 203 |
| | - | - | 300 | - | - | - | - | - | - | - | - | 205 |
| | DNW | - | - | - | DNW | - | - | - | - | - | - | - |
| | - | - | - | - | - | - | - | - | - | - | - | 205 |
| | DNW | - | - | - | - | - | - | - | - | - | DNW | DNW |
| | 236 | - | - | - | - | - | - | - | - | - | - | - |
| | DNW | - | - | - | DNW | - | - | - | - | - | - | - |
| | 236 | - | - | - | - | - | - | - | - | - | - | - |
| | 236 | - | 318 | - | - | - | - | - | - | - | - | - |
| | DNW | - | - | - | DNW | - | - | - | - | - | - | - |
| | 236 | - | 300 | - | - | - | - | - | - | - | - | - |
| | 236 | - | - | - | - | - | - | - | - | - | - | DNW |
| | DNW | - | 300 | - | DNW | DNW | - | - | - | - | - | - |
| | DNW | - | 307 | - | DNW | - | - | - | - | - | - | - |
| | DNW | - | 318 | - | - | - | - | - | - | - | - | - |
| | DNW | DNW | DNW | - | - | - | - | - | - | 147 | DNW | DNW |
| | DNW | DNW | DNW | - | - | - | - | - | - | - | DNW | DNW |
| C580Y WESTERN THAILAND | DNW | - | - | - | 278 | - | - | - | - | DNW | DNW | - |
| | - | 198 | - | - | 278 | 117 | - | - | - | - | - | - |
| | DNW | - | 300 | - | DNW | - | - | - | - | DNW | - | - |
| | DNW | 198 | - | - | DNW | DNW | - | - | - | - | - | - |
| | - | 198 | - | - | 278 | - | - | - | - | - | - | - |
| | - | 198 | - | - | 278 | - | - | - | - | - | - | - |
| | DNW | - | - | - | 278 | - | - | - | 284 | DNW | - | - |
| | DNW | 198 | - | - | 278 | - | - | - | - | 150 | - | 205 |
| | DNW | 198 | - | - | 278 | - | - | - | DNW | - | DNW | - |
| | DNW | 198 | - | - | 278 | DNW | - | - | - | DNW | - | - |

## FLANKING K13 MS (WILD TYPE)

| Group | 72.3kb | 31.5kb | 31.0kb | 15.1kb | 8.6kb | 3.4kb | -0.15kb | -3.74kb | -6.36kb | -31.9kb | -50.0kb | -56.0kb |
|---|---|---|---|---|---|---|---|---|---|---|---|---|
| EASTERN THAILAND | 248 | 194 | 310 | 138 | 288 | 131 | 193 | 148 | 282 | 201 | 258 | 203 |
| | 232 | - | 309 | - | DNW | - | - | - | DNW | DNW | - | - |
| | 232 | DNW | DNW | - | - | - | - | 150 | - | DNW | DNW | DNW |
| | 248 | - | - | 144 | DNW | 121 | 197 | 150 | 268 | DNW | - | 205 |
| | 244 | - | 305 | - | 278 | - | - | - | - | DNW | - | 205 |
| | DNW | 198 | - | 144 | 280 | 121 | 197 | 150 | 268 | - | DNW | DNW |
| | 240 | - | - | - | DNW | - | - | - | - | - | - | - |
| | DNW | 204 | 305 | - | 266 | 123 | - | - | - | - | - | - |
| | 236 | - | - | - | 282 | 125 | 197 | 150 | 285 | 208 | - | - |
| | DNW | 198 | - | - | - | - | 197 | 154 | 288 | 216 | - | 213 |
| | - | - | - | - | - | - | - | - | - | - | - | - |
| | DNW | - | - | - | DNW | - | - | - | DNW | - | - | 205 |
| | DNW | DNW | - | 144 | 280 | 121 | 197 | 150 | 268 | DNW | DNW | DNW |
| | DNW | - | 305 | - | - | - | - | - | - | DNW | - | - |
| EASTERN THAILAND | 238 | 204 | - | - | - | - | 197 | 150 | 274 | 204 | - | 205 |
| | DNW | 198 | - | - | - | 119 | - | - | 266 | - | - | - |
| | DNW | - | 300 | - | 278 | - | - | - | - | - | - | - |
| | DNW | 198 | - | - | DNW | - | - | - | DNW | DNW | - | - |
| | DNW | 198 | 300 | - | - | 115 | - | - | - | 218 | - | - |
| | 238 | DNW | DNW | - | - | 129 | - | - | 266 | DNW | - | DNW |
| | DNW | 204 | 300 | - | - | - | 197 | 150 | 274 | - | - | - |
| | - | DNW | DNW | - | 278 | - | - | - | - | 145 | - | DNW |
| | DNW | DNW | DNW | - | 278 | 119 | 197 | 150 | 274 | DNW | DNW | DNW |
| | DNW | 198 | DNW | - | DNW | - | - | - | DNW | - | - | - |
| | - | DNW | - | - | 278 | - | - | - | - | - | DNW | DNW |
| | 244 | DNW | - | - | 273 | - | DNW | 150 | DNW | DNW | DNW | DNW |
| | DNW | DNW | DNW | - | - | - | 197 | 150 | 274 | 204 | - | DNW |
| | 244 | DNW | DNW | - | - | - | - | - | - | DNW | DNW | DNW |
| | DNW | DNW | DNW | - | 278 | - | - | - | - | - | - | DNW |
| | - | 190 | - | - | DNW | - | - | - | - | DNW | DNW | DNW |
| | 238 | - | - | - | DNW | - | - | - | - | - | - | - |
| | 238 | - | 300 | - | 278 | - | - | - | DNW | 141 | - | - |
| | 240 | 198 | 300 | - | - | - | - | - | - | - | DNW | DNW |
| | DNW | 202 | 295 | - | - | - | - | - | - | - | DNW | DNW |
| | 242 | 198 | 300 | - | - | 115 | - | - | - | - | - | - |
| | DNW | - | 300 | - | 278 | 119 | 197 | 150 | 274 | 204 | - | - |
| | DNW | - | - | - | DNW | 115 | - | - | DNW | - | - | - |
| | DNW | - | 300 | - | - | - | - | 150 | 280 | 207 | - | - |
| | 246 | DNW | DNW | - | - | - | - | 150 | 280 | DNW | DNW | DNW |
| | 244 | 198 | - | - | 278 | - | - | - | - | 205 | - | - |
| | - | DNW | 300 | - | - | - | - | - | - | 211 | DNW | DNW |
| | DNW | - | DNW | - | 278 | - | - | - | - | - | - | - |
| | - | 198 | - | 144 | DNW | 112 | - | 150 | 271 | DNW | - | - |
| | 238 | - | - | - | DNW | - | - | - | - | - | - | - |
| | 250 | 198 | DNW | - | DNW | 115 | - | - | - | 204 | DNW | DNW |
| | DNW | 198 | DNW | - | - | 115 | - | - | - | DNW | - | - |
| | 238 | 198 | - | - | - | 115 | - | - | - | 218 | - | - |
| | 244 | 198 | - | - | - | - | - | - | - | 210 | - | - |
| | 236 | 198 | 272 | - | 293 | 129 | - | - | 266 | 210 | - | - |
| | DNW | - | - | - | 278 | - | - | - | - | 210 | - | 205 |
| | DNW | - | - | 144 | DNW | 113 | - | - | 277 | 208 | - | - |
| | 238 | 198 | - | 144 | 278 | - | - | - | - | 210 | - | 205 |
| | DNW | 198 | DNW | - | DNW | - | - | - | - | - | - | 205 |
| | 250 | - | - | - | 278 | - | - | - | - | - | - | 201 |
| | 250 | - | - | - | 280 | 121 | 191 | DNW | DNW | 206 | DWN | - |
| | DNW | 198 | - | - | DNW | 127 | - | 157 | DNW | DNW | DNW | 205 |
| | DNW | DNW | DNW | - | - | 140 | 190 | - | 280 | - | DNW | DNW |
| | DNW | 198 | DNW | - | - | 115 | - | - | DNW | DNW | DNW | 205 |
| | DNW | DNW | DNW | - | 278 | 119 | 197 | 150 | 274 | 209 | DNW | DNW |
| | DNW | 198 | 300 | - | - | 115 | - | - | - | 218 | - | - |
| | DNW | - | - | - | DNW | - | - | - | DNW | - | - | - |
| | DNW | DNW | DNW | - | 266 | 123 | - | 150 | 274 | DNW | DNW | DNW |
| | DNW | DNW | DNW | - | DNW | 129 | - | 144 | 268 | DNW | DNW | DNW |
| | DNW | DNW | DNW | - | - | - | - | 150 | 280 | DNW | DNW | DNW |
| | DNW | DNW | DNW | - | 297 | 127 | - | 155 | 277 | DNW | DNW | DNW |
| | DNW | DNW | DNW | 141 | 284 | 127 | 197 | 153 | 280 | DNW | DNW | DNW |
| | 236 | DNW | DNW | - | 278 | - | - | - | - | - | DNW | DNW |
| | DNW | 202 | - | - | DNW | - | 197 | 150 | DNW | 135 | - | - |
| | 236 | 198 | - | - | - | 119 | 197 | 150 | 277 | 141 | - | 205 |
| | - | 198 | - | - | DNW | 119 | 197 | 150 | 277 | DNW | - | 205 |
| | 250 | 198 | - | - | DNW | DNW | 197 | 150 | 277 | DNW | - | 205 |
| | DNW | 198 | - | - | DNW | 119 | 197 | 150 | 277 | DNW | - | 205 |
| | 240 | 198 | - | - | DNW | DNW | 197 | 150 | 277 | DNW | DNW | DNW |

| **Province** | **Number of samples** |
|---|---|
| Mae Hon Son | 42 |
| Tak | 171 |
| Kanchanaburi | 40 |
| Prachuap | 33 |
| Ranong | 40 |
| Chumporn | 12 |
| Sisaket | 13 |
| Chanthaburi | 10 |
| Trat | 12 |
| Yala | 44 |
| **Total** | **417** |
