# Supplementary figures and images for: Selection and Spread of Artemisinin-Resistant Alleles in Thailand Prior to the Global Artemisinin Resistance Containment Campaign
Source: PLoS Pathog. 2015 Apr 2;11(4):e1004789. doi: 10.1371/journal.ppat.1004789 (PMC4383523; doi:10.1371/journal.ppat.1004789)

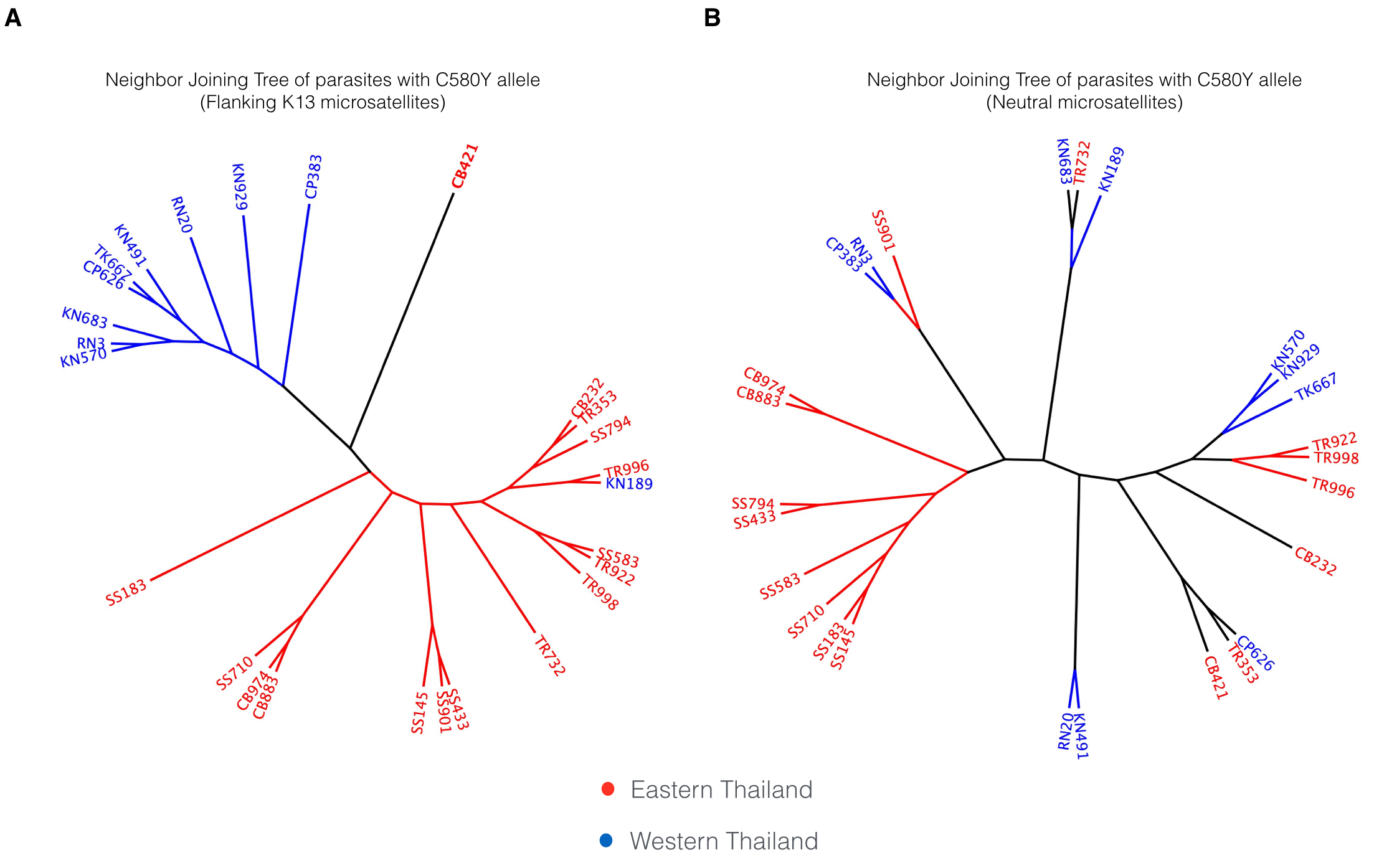

Supplement: S1 Fig — Relationships among parasites with the C580Y allele, based on K13 flanking microsatellites (A) and neutral microsatellites (B). Provinces are abbreviated as follows: in the east, Chanthaburi (CB), Trat (TR), and Sisaket (SS); in the west, Tak (TK), Kanchanaburi (KN), Chumporn (CP), Ranong (RN). Red branches indicate parasite isolates from eastern Thailand (CB, TR, SS) and blue branches, parasites from western Thailand (TK, KN, CP, and RN). (TIF) [file ppat.1004789.s002.tif]
