# Supplementary material for: Selection and Spread of Artemisinin-Resistant Alleles in Thailand Prior to the Global Artemisinin Resistance Containment Campaign
Source: PLoS Pathog. 2015 Apr 2;11(4):e1004789. doi: 10.1371/journal.ppat.1004789 (PMC4383523; doi:10.1371/journal.ppat.1004789)
Supplement: S1 Table — (PDF) [file ppat.1004789.s003.pdf]

Table 1

| | | |
|---|---|---|
| | A total of 25 MS markers were tested flanking the K13 gene. In **bold** are MS markers (8.6kb and 31.5kb) that were informative and separated the C580Y haplotypes by geography. In green are MS markers = successful amplification. *Please refer to Table S1 for final raw data.* | |
| **Number** | Locus | Primer seq (5' to 3') |
| **1** | 0.1kb | ATGAAGAAATTTTAAATGTTC |
| | | ATTTATAGCTAATAAGTAATATC |
| **2** | 0.5kb | TATGATAATATAAATTCTCATG |
| | | TATTAATAGAATTGTTCAAACAT |
| **3** | 0.1kb | CTTTAATATTTATTTTCATGCAC |
| | | GAACAATTCTATTAATATTTTAAG |
| **4** | 3.4kb | TCTACAGCGGTAAGATGAATAG |
| | | TCCTTGGCATGTCCCGGCTGC |
| **5** | **8.6kb** | **ACGGGTTTCTGTACGCCTCAC** |
| | | **CTAAATTAGTGGCTTCTTCAT** |
| **6** | 15.1kb | CTTACTTCTAGGAGAATCCT |
| | | GATAACTACTCATCTCCACA |
| **7** | 20.0kb | CTAAACCAAATGAGATAAGG |
| | | GTTATACTATTATACATATGTATAGG |
| **8** | 21.0kb | TCCTATACATATGTATAATAGTATAAC |
| | | AAGGATTCTTCGTACCTATG |
| **9** | 31.0kb | GAAGATGTAGATATTACTTGTA |
| | | TATAAGATTCGTTATTACATG |
| **10** | **31.5kb** | **GTACAAATGAACCTCTTCC** |
| | | **AATAATTCACCATGTCATGC** |
| **11** | 42.0kb | TGTTAAGCATCCATTCTCTC |
| | | CATCTTAAATGTACAAATATGTCC |
| **12** | 43.0kb | CATCTTAAATGTACAAATATGTCC |
| | | TGTTAAGCATCCATTCTCTC |
| **13*** | 72.3kb | TGTTAAGCATCCATTCTCTC |
| | | TGTTAAGCATCCATTCTCTC |
| **14** | -0.1kb | ACAAGGCGTAAATATTCGTGT |
| | | GATTCCCTATCATACGTCATAG |
| **15** | -0.15kb | GATTCCCTATCATACGTCATAG |
| | | ACAAGGCGTAAATATTCGTG |
| **16** | -0.4kb | CGAAAAAATAATTTATTATTCATAC |
| | | TATATATACATTTACATATGG |
| **17** | -2.5kb | TGAAAACATATACATAGAATG |
| | | TTCACAAATATATCACTGC |
| **18** | -2.0kb | GCAGTGATATATTTGTGAACAG |
| | | TCCTTCATATTACATAAAGTCC |
| **19** | -3.0kb | GTTAATTATTGGGCTTACATTC |
| | | GTCAATATTTATAATTTAGATGAG |
| **20** | -3.74kb | GTCAATATTTATAATTTAGATGAG |
| | | GTCAATATTTATAATTTAGATGAG |
| **21** | -6.36kb | AATTAGACAGGACTTGTTAATG |
| | | GGATATGTTATCCAAAATGCA |
| **23*** | -31.9kb | AATCAATCATGGAAATTATG |
| | | TTGTTCATTTAAGGGTATTT |
| **24** | -36.0kb | ACATATTATTATGTGTGGATGTC |
| | | ATAACAAATCAATAAATGAACTC |
| **25** | -40.0kb | TTAACGAGAATCGCTTCAT |
| | | TAATTATCACTTAATTATATTG |
| **26** | -45.5kb | AGCACAATAAAGTTGGC |
| | | TCTACCAGTATTAATATTTGAG |
| **27** | -50.0kb | ATGACGAGGATAACCATG |
| | | AATATGTACGATCATTACAGT |
| **28** | -56.0kb | TCCTTCTATTTGTGTGAGTACAG |
| | | TATTGTAATGCATGCACTG |
| | | *Cheeseman, I.H., et al., A major genome region underlying artemisinin resistance in malaria. Science, p. 79-82. 2012. 336(6077): |
